# Occurrence of disputed *rpoB* mutations among *Mycobacterium tuberculosis* isolates phenotypically susceptible to rifampicin in a country with a low incidence of multidrug-resistant tuberculosis

**DOI:** 10.1186/s12879-018-3638-z

**Published:** 2019-01-03

**Authors:** Noura M. Al-Mutairi, Suhail Ahmad, Eiman Mokaddas, Hanaa S. Eldeen, Susan Joseph

**Affiliations:** 1 Department of Microbiology, Faculty of Medicine, Health Sciences Centre, Kuwait University, P. O. Box 24923, 13110 Safat, Kuwait; 2 Kuwait National TB Control Laboratory, Shuwaikh, Kuwait

**Keywords:** *Mycobacterium tuberculosis*, Polydrug resistance, *rpoB* disputed mutations, Prevalence

## Abstract

**Background:**

Accurate drug susceptibility testing (DST) of *Mycobacterium tuberculosis* in clinical specimens and culture isolates to first-line drugs is crucial for diagnosis and management of multidrug-resistant tuberculosis (MDR-TB). Resistance of *M. tuberculosis* to rifampicin is mainly due to mutations in hot-spot region of *rpoB* gene (HSR-*rpoB*). The prevalence of disputed (generally missed by rapid phenotypic DST methods) *rpoB* mutations, which mainly include L511P, D516Y, H526N, H526L, H526S, and L533P in HSR-*rpoB* and I572F in cluster II region of *rpoB* gene, is largely unknown. This study determined the occurrence of all disputed mutations in HSR-*rpoB* and at *rpoB* codon 572 in *M. tuberculosis* strains phenotypically susceptible to rifampicin in Kuwait.

**Methods:**

A total of 242 *M. tuberculosis* isolates phenotypically susceptible to rifampicin were used. The DST against first-line drugs was performed by Mycobacteria growth indicator tube (MGIT) 960 system. Mutations in HSR-*rpoB* (and *katG* codon 315 and *inhA*-regulatory region for isoniazid resistance) were detected by GenoType MDBDR*plus* assay. The I572F mutation in cluster II region of *rpoB* was detected by developing a multiplex allele-specific (MAS)-PCR assay. Results were confirmed by PCR-sequencing of respective loci. Molecular detection of resistance for ethambutol and pyrazinamide and fingerprinting by spoligotyping were also performed for isolates with an *rpoB* mutation.

**Results:**

Among 242 rifampicin-susceptible isolates, 0 of 130 pansusceptible/monodrug-resistant isolates but 4 of 112 polydrug-resistant isolates contained a disputed *rpoB* mutation. All 4 isolates were also resistant to isoniazid and molecular screening identified additional resistance to pyrazinamide and ethambutol in one isolate each. In final analysis, 2 of 4 isolates were resistant to all 4 first-line drugs. Spoligotyping showed that the isolates belonged to different *M. tuberculosis* lineages.

**Conclusions:**

Four of 242 (1.7%) rifampicin-susceptible *M. tuberculosis* isolates contained a disputed *rpoB* mutation including 2 isolates resistant to all four first-line drugs. The occurrence of a disputed *rpoB* mutation in polydrug-resistant *M. tuberculosis* isolates resistant at least to isoniazid (MDR-TB) suggests that polydrug-resistant strains should be checked for genotypic rifampicin resistance for optimal patient management since the failure/relapse rates are nearly same in isolates with a canonical or disputed *rpoB* mutation.

## Background

Widespread occurrence of drug-resistant tuberculosis (TB) and multidrug-resistant (MDR)-TB (infection with *Mycobacterium tuberculosis* strain resistant at least to rifampicin, RIF and isoniazid, INH; the two most effective first-line anti-TB drugs) is a serious threat to TB control success worldwide. According to global annual surveys conducted by World Health Organization (WHO), an estimated 490,000 cases of MDR-TB occurred among 10.4 million new active TB cases in 2016 [1]. Compared to drug-susceptible TB, treatment of MDR-TB is more expensive, drug regimens are more toxic and require longer (18–24 months) duration of treatment which often results in clinical failure or disease relapse [1–3]. Unsuccessful treatment of MDR-TB is a risk factor for extensively drug-resistant TB (XDR-TB, infection with MDR-TB strains additionally resistant to a fluoroquinolone and injectable agent such as kanamycin, amikacin or capreomycin) which is often fatal in developing countries [2–4]. Accurate drug susceptibility testing (DST) of *M. tuberculosis* in clinical specimens and culture isolates to first-line drugs is crucial for rapid diagnosis of MDR-TB for proper patient management, for limiting further transmission of MDR-TB and development of XDR-TB [2, 5]. Although rapid liquid culture-based phenotypic DST methods are considered as the gold standard by WHO for identifying resistance to RIF, INH and other first-line drugs, these methods still require 1–2 weeks to report results [5, 6]. Molecular DST methods rapidly detect genetic mutations associated with drug resistance [2, 7].

Resistance of *M. tuberculosis* to RIF in 95–97% isolates is due to mutations in an 81-base pair (bp) hot-spot region (HSR) of the *rpoB* gene (HSR-*rpoB*) [8]. The remaining 3–5% isolates contain mutations in N-terminal or cluster II region of the *rpoB* gene or in other genes [8, 9]. *M. tuberculosis* isolates with canonical (undisputed) HSR-*rpoB* mutations (like Q513P, Q513K, H526R, S531 L or S531 W, *Escherichia coli* numbering system, [8]) as well as isolates with mutations (such as V146F) in the N-terminal end of the *rpoB* gene exhibit high-level resistance to RIF which are readily detected by rapid phenotypic DST methods [8, 9]. Some molecular assays targeting HSR-*rpoB* are not specific as silent mutations in this region may occasionally lead to detection of false-positive RIF resistance [10]. Recent studies have also shown that rapid liquid culture systems such as Mycobacteria growth indicator tube (MGIT) 960 system as well as the proportion method with shorter (4 weeks) incubation time often fail to detect strains exhibiting low-level (minimum inhibitory concentration, MIC of 0.5–2.0 μg/ml) resistance to RIF [11–14]. These low-level RIF-resistant strains with increased MICs below the critical concentration mostly contain specific mutations within HSR-*rpoB*, particularly at codon 511 (such as L511P), codon 516 (such as D516Y), codon 526 (such as H526N, H526L and H526S), and codon 533 (such as L533P) [11–14]. Mutation I572F in cluster II region of the *rpoB* gene also increases MICs below the critical concentration conferring low-level resistance to RIF [11–14]. Nearly 30% RIF-resistant *M. tuberculosis* isolates from Swaziland contained this (disputed) mutation and rapid liquid culture systems failed to accurately detect strains with this mutation [15]. The clinical significance of some (D516Y and I572F) of these disputed (generally missed by rapid phenotypic DST methods) mutations in conferring resistance to RIF is indicated by gene replacement studies [16]. Low-level resistance to RIF is clinically significant as patients infected with *M. tuberculosis* strains with disputed *rpoB* mutations often fail treatment or relapse [17–20]. The prevalence of *M. tuberculosis* isolates with disputed *rpoB* mutations is largely unknown since phenotypic DST in low TB incidence, high income countries is usually carried out by rapid liquid culture-based methods. This study determined the occurrence of disputed mutations in HSR-*rpoB* as well as I572F mutation in cluster II region of the *rpoB* gene in clinical *M. tuberculosis* strains phenotypically susceptible to RIF in Kuwait, a country with low (24 per 100,000) incidence of TB as well as a low (~ 1%) incidence of MDR-TB [1, 21]. Common mutations conferring resistance to INH were also detected [7, 9]. For isolates with an *rpoB* mutation, molecular detection of resistance for two other first-line drugs for which rapid culture-based DST methods are either cumbersome (pyrazinamide, PZA) [5, 6, 22, 23] or unreliable (ethambutol, EMB) [5, 6, 24–26] was also performed.

## Methods

### *M. tuberculosis* isolates

A total of 242 *M. tuberculosis* isolates phenotypically susceptible to RIF were selected from our culture collection. The isolates were grown from 144 pulmonary (sputum, *n* = 131 and BAL, 13) and 98 extrapulmonary (fine needle aspirate and pus, *n* = 66; pleural fluid, *n* = 11; lymph node, *n* = 8; tissue, *n* = 7; cerebrospinal fluid, *n* = 4 and gastric aspirate, *n* = 2) specimens collected from 242 suspected TB patients as part of routine patient care at Kuwait National TB Reference Laboratory. The samples were processed for culture on solid (Lowenstein-Jensen) and liquid media-based automated MGIT 960 system. All the patients were newly diagnosed active TB disease cases and the isolates were cultured before initiation of anti-TB treatment. Data analyses were carried out on deidentified results.

## Culture and drug susceptibility testing by MGIT 960 system

Non-sterile clinical specimens were processed by the standard *N*-acetyl-L-cysteine and sodium hydroxide (NALC/NaOH) method while sterile samples were processed directly [21]. The NALC (0.5%) was used to digest the sputum specimens while NaOH (4%) and sodium citrate (2.94%) were used to decontaminate the sample. All specimens were cultured on solid (Lowenstein-Jensen) and MGIT 960 system media according to the manufacturer’s instructions and as described previously [21, 26]. The MGIT 960 system cultures were incubated for at least 4 weeks and cultures flagged positive for growth were used for the extraction of DNA by the rapid Chelex-100-based method, as described previously [27]. The presence of *M. tuberculosis* complex DNA was detected by AccuProbe DNA probe assay and an in-house multiplex PCR assay, as described previously [21, 28]. The MGIT 960 system cultures were also subjected to phenotypic DST by using the SIRE drug kit (Becton Dickinson, Sparks, MD, USA), which contains streptomycin (SM) at 1.0 μg/ml, INH at 0.1 μg/ml, RIF at 1.0 μg/ml, and EMB at 5.0 μg/ml, as described previously [21, 26]. The tubes were incubated in the MGIT 960 system for at least 2 weeks or until the results indicating susceptibility or resistance were automatically interpreted and reported by using predefined algorithms which compared the growth in the drug-containing tube with the growth in control tube. Phenotypic DST for PZA was also performed on selected isolates, by using MGIT 960 PZA kit (Becton Dickinson) according to the manufacturer’s instructions.

## Detection of mutations conferring resistance to anti-TB drugs

All mutations (including disputed mutations) in HSR-*rpoB* in the DNA samples were detected by GenoType MDBDR*plus* assay as described previously [29]. This assay also simultaneously detects mutations at *katG* codon 315 (*katG315*) and *inhA* regulatory region (*inhA*-RR) which confer resistance to INH [29]. The V146F mutation in the N-terminal region (outside the hot-spot region) of the *rpoB* gene was not targeted as this mutation confers high-level resistance to rifampicin [30]. However, the I572F mutation in cluster II region of *rpoB* gene (also outside the hot-spot region) was targeted for detection in this study as this mutation confers low-level resistance to rifampicin and is frequently missed by rapid phenotypic methods [11–13]. The I572F mutation in the *rpoB* gene was detected by developing a multiplex allele-specific (MAS)-PCR assay.

## Development of MAS-PCR assay for detection of I572F mutation in *rpoB* gene

The detection of I572F mutation in cluster II region of *rpoB* gene was achieved by developing a MAS-PCR assay. For this purpose, three oligonucleotide primers (NArpoBF, 5’-TCATGGACCAGAACAACCCGCTGT-3′; NArpoBR, 5’-GTACGGCGTTTCGATGAACCCGAA-3′ and NArpoB572F, 5’-GGGCCCAACATCGGTCTGTT-3′) were designed. MAS-PCR reactions in a final volume of 50 μl contained 1x AmpliTaq PCR buffer I, 1 U AmpliTaq DNA polymerase, 8 pmol of NArpoBF primer, 8 pmol of NArpoB572F primer, 16 pmol of NArpoBR primer, 2 μl of DNA and 0.1 mM of each dNTP. Touchdown PCR cycling conditions were same and detection of amplicons on 2% agarose gels was performed, as described previously [27]. The MAS-PCR should yield only one amplicon of 232 bp from reference strain *M. tuberculosis* H_37_Rv and clinical isolates containing isoleucine at codon 572 (I572, wild-type sequence) but two amplicons of 232 bp and 78 bp from isolates containing phenylalanine at codon 572 (I572F mutation). The results for all isolates with an *rpoB* mutation and for isolates with mutations at *katG315* and/or *inhA*-RR indicated only by lack of hybridization with a wild-type probe were confirmed by PCR-sequencing of respective loci, performed as described previously [29].

## Detection of mutations conferring resistance to EMB and PZA

Common mutations conferring resistance to EMB (in *embB* gene) and PZA (in *pncA* gene) [2, 9] were also detected among *M. tuberculosis* isolates carrying disputed *rpoB* mutations. Mutations conferring resistance to EMB were detected by PCR-sequencing of *embB* gene as described previously [26]. Mutations conferring resistance to PZA were detected by PCR amplification and DNA sequencing of two overlapping *pncA* fragments. The N-terminal fragment was amplified by using PNCANF (5’-GCGTCATGGACCCTATATCT-3′) and PNCANR (5’-TTCGAAGCCGCTGTACGCTC-3′) while the C-terminal fragment was amplified by using PNCACF (5’-TCCATCCCAGTCTGGACACG-3′) and PNCACR (5’-GCGCGTCACCGGTGAACAAC-3′) primers and the touchdown PCR protocol described previously [27]. The amplicons were purified and both strands were sequenced by using the same amplification primers and the sequencing protocol described previously [29]. The nucleotide and deduced amino acid sequences were compared with the corresponding sequences from susceptible strain *M. tuberculosis* H_37_Rv by using Clustal Omega.

## Spoligotyping

The isolates with an *rpoB* mutation were also subjected to spoligotyping to see if the isolates that have disputed mutation are clustered together. The procedure was performed by using the SPOLIGO TB kit (Mapmygenome India Limited, Hyderabad, India) according to kit instructions and as described previously [31]. In brief, the 43 spacers within direct repeat region in *M. tuberculosis* isolates were amplified by using biotinylated Dra primer together with Drb primer, PCR products were hybridized to 43 spacer oligonucleotides embedded on Spoligo-membrane and the hybridization signals were detected by enhanced chemiluminescence. The detected bands were converted to 43 binary codes which were used for assignment of phylogenetic lineages according to SITVIT database (http://www.pasteur-guadeloupe.fr:8081/SITVITDemo/index.jsp). Quality control was ensured by using *M. tuberculosis* H_37_Rv and *M. bovis* BCG P3 as positive controls.

## Statistical analysis

Categorical variables were expressed as absolute number. Statistical analysis was performed by using chi-square test or Fisher’s exact test as appropriate and probability levels < 0.05 by the two-tailed test were considered as statistically significant. Statistical analyses were performed by using WinPepi software ver. 11.65 (PEPI for Windows, Microsoft Inc., Redmond, WA, USA).

## Results

### Phenotypic DST data by MGIT 960 system

Phenotypic DST by MGIT 960 system showed that 64 isolates were fully susceptible to all four (RIF, INH, SM and EMB) anti-TB drugs (pansusceptible strains), 15 isolates were resistant to SM only, 51 isolates were resistant to INH only while the remaining isolates were resistant to more than one drug (polydrug-resistant strains). Among 112 polydrug-resistant strains, 75 isolates were resistant to INH + SM, 26 isolates were resistant to INH + EMB and 11 isolates were resistant to three (INH + SM + EMB) drugs (Table 1).

**Table 1:** Phenotypic resistance by MGIT 960 system to anti-TB drugs and genotypic screening of mutations in HSR-*rpoB*, *katG315* and *inhA-*RR among 242 clinical *M. tuberculosis* isolates

Phenotypic resistance to anti-TB drug	No. of isolates tested	Number of isolates detected by gMTBDR^+^ with a mutation in		
		HSR-*rpoB*	*katG315* (S315 T)	*inhA*-RR
None	64	0	0	0
SM	15	0	0	0
INH	51	0	15	21^a^
INH + SM	75	2	58	11^b^
INH + EMB	26	1	10	2^b^
INH + SM + EMB	11	1	6	0

gMTBDR^+^, GenoType MTBDRplus assay; HSR-*rpoB*, 81-base pair hot-spot region of *rpoB* gene; *katG315* (S315 T), S315 T mutation at *katG* codon 315; *inhA*-RR, upstream regulatory region of *inhA* gene

*SM* streptomycin, *INH* isoniazid, *EMB* ethambutol

^a^20 isolates contained − 15 C/T and 1 isolate contained − 8 T/A mutation

^b^All isolates contained − 15 C/T mutation

### Detection of mutations in *rpoB*, *katG* and *inhA* genes

Mutations in HSR-*rpoB*, *katG315* and *inhA*-RR were detected by the GenoType MDBDR*plus* assay and/or PCR-sequencing. The data showed that all pansusceptible (*n* = 64) and SM-monoresistant (*n* = 15) isolates contained wild-type HSR-*rpoB*, *katG315* and *inhA*-RR sequences (Table 1). All INH-monoresistant isolates (*n* = 51) also contained wild-type HSR-*rpoB* sequences, however, 15 (30%) isolates contained *katG315* (S315 T) mutation while 20 (41%) isolates contained − 15 C/T and 1 isolate contained − 8 T/G mutation in *inhA*-RR (Table 1). Among 75 isolates with phenotypic resistance to INH + SM, 2 isolates contained a mutation in HSR-*rpoB* (both isolates contained H526N mutation), 58 (77%) isolates (including both isolates with H526N mutation) contained *katG315* (S315 T) mutation and 11 (15%) isolates contained − 15 C/T *inhA*-RR mutation (Table 1). Among 26 isolates with phenotypic resistance to INH + EMB, 1 isolate contained D516Y mutation in HSR-*rpoB*, 10 (39%) isolates (including the isolate with D516Y mutation) contained *katG315* (S315 T) mutation and 2 (8%) isolates contained − 15 C/T *inhA*-RR mutation (Table 1). Furthermore, among 11 isolates with phenotypic resistance to INH + SM + EMB, 1 isolate contained S531C mutation in HSR-*rpoB*, 6 (55%) isolates (including the isolate with S531C mutation) contained *katG315* (S315 T) mutation while no isolate contained a mutation in *inhA*-RR (Table 1). Mutation I572F in cluster II region of the *rpoB* gene was detected by developing a MAS-PCR assay.

### Development of multiplex allele-specific (MAS)-PCR for detection of I572F mutation

**Fig. 1:**
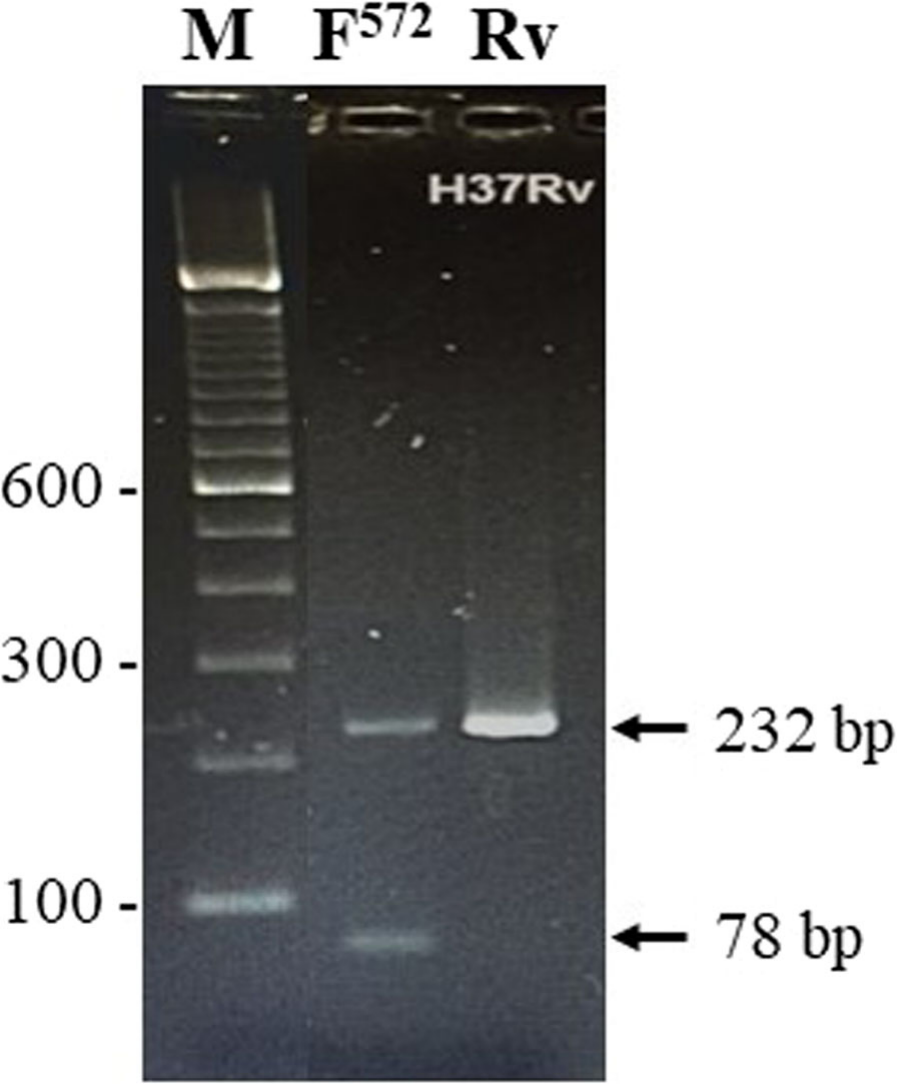
Agarose gel of multiplex allele-specific (MAS)-PCR amplicons obtained from *M. tuberculosis* H_37_Rv containing wild-type (I572) sequence (lane Rv) and rifampicin-resistant isolate 5177/06 containing I572F mutation (lane F^572^) at *rpoB* codon 572 obtained with NArpoBF, NArpoBR, and NArpoB572F primers. The position of migration of 232 bp and 78 bp fragments are indicated by arrows. Lane M is 100 bp DNA ladder and the positions of migration of 100 bp, 300 bp and 600 bp fragments are marked

The multiplex allele-specific (MAS)-PCR developed in this study accurately detected the presence of wild-type (I572) sequence at *rpoB* codon 572 in reference strain, *M. tuberculosis* H_37_Rv and presence of I572F mutation in a well characterized clinical isolate (5177/06) containing this mutation (Fig. 1). The isolate 5177/06 was detected as RIF-susceptible by MGIT 960 system [29]. When MAS-PCR assay was performed on 10 selected pansusceptible and all 112 polydrug-resistant isolates, the data showed the presence of wild-type sequence (I572) at *rpoB* codon 572 in each isolate. Thus, I572F mutation was not detected and all disputed mutations were detected only in HSR-*rpoB* in this study. Furthermore, all isolates with a disputed *rpoB* mutation were polydrug-resistant strains.

Altogether, 4 of 242 (1.7%) isolates phenotypically susceptible to RIF contained a disputed *rpoB* mutation, were also resistant to INH (containing the common S315 T mutation at *katG315*) and so should be classified as MDR-TB strains. The occurrence of a disputed *rpoB* mutation varied from 4 of 112 (3.6%) in polydrug-resistant isolates resistant at least to INH to 3 of 101 (3.0%) in isolates resistant to INH + one more drug. The relative occurrence of a disputed mutation was higher (1 of 11, 9.1%) in isolates resistant to 3 (INH + SM + EMB) drugs compared to 2 drugs (3 of 101, 3%). Also, the occurrence of a disputed *rpoB* mutation was higher in INH-resistant isolates additionally resistant to EMB compared to INH-resistant isolates additionally resistant to SM (2 of 37, 5.4% versus 3 of 86, 3.5%), however, the difference was not statistically significant. Another interesting observation was the decreasing proportion of *inhA-*RR mutations in INH-resistant *M. tuberculosis* isolates with additional resistance to increasing number of other (SM and/or EMB) drugs (Table 1). The proportion of *inhA-*RR mutations in INH-monoresistant isolates (21 of 51, 41%) was significantly higher compared to INH-resistant isolates additionally resistant to one more drug (13 of 101, 13%) (*p* = 0.000) or two more drugs (0 of 11, 0%) (*p* = 0.011).

### Mutations in *embB* and *pncA* in *M. tuberculosis* isolates with *rpoB* mutations

Further genotypic characterization of the 4 discrepant isolates (denoted as D1 to D4) containing a disputed *rpoB* mutation is shown in Table 2. PCR-sequencing of *embB* gene showed that both isolates phenotypically resistant to EMB as well as 1 isolate (D3) phenotypically susceptible to EMB contained M306 V mutation in *embB* which confers low-level resistance to EMB [16, 25]. Phenotypic DST data for PZA was available for 2 isolates only due to technical difficulties; 1 isolate (D3) was resistant while the other isolate (D4) was susceptible. The PZA-resistant isolate contained the well-characterized H51P mutation [22, 23] while the PZA-susceptible isolate contained wild-type *pncA* sequence. Another isolate (D2) for which phenotypic PZA susceptibility data was not available (due to lack of growth at lower pH) contained a non-synonymous (R2P) mutation in *pncA*. Thus, based on molecular screening, isolates D1 and D4 were additionally resistant to RIF, isolate D2 was additionally resistant to RIF and PZA while isolate D3 was additionally resistant to RIF and EMB (Table 2). Spoligotyping performed on all 4 isolates showed that they belonged to different *M. tuberculosis* lineages (Table 2).

**Table 2:** Phenotypic susceptibility and genotypic characterization of *M. tuberculosis* isolates with disputed *rpoB* mutations

Isolate	Culture	Clinical	Phenotypic resistance	Susceptibility	Mutations detected in^c^			*embB*	*pncA*	Spoligotyping	Final resistance
no.	no.	specimen	to anti-TB drugs^b^	to PZA^b^	*rpoB*	*katG315*	*inhA-*RR	mutation	mutation	lineage	pattern
D1	5853/05	BAL^a^	INH + SM	Not done	H526N	S315 T	None	None	None	Orphan	INH + SM + RIF
D2	13,242/10	Sputum	INH + EMB	Not done	D516Y	S315 T	None	M306 V	R2P^d^	EAI5/EAI3	INH + EMB + RIF + PZA
D3	10,268/11	Pleural fluid	INH + SM	Resistant	H526N	S315 T	None	M306 V	H51P	Orphan	INH + SM + PZA + RIF + EMB
D4	13,341/16	Sputum	INH + SM + EMB	Susceptible	S531C	S315 T	None	M306 V	None	T1 Uganda	INH + SM + EMB + RIF

INH, isoniazid; SM, streptomycin; EMB, ethambutol; RIF, rifampicin, PZA, pyrazinamide

HSR-*rpoB*, 81-base pair hot-spot region of *rpoB* gene; *katG315*, *katG* codon 315; *inhA*-RR, upstream regulatory region of *inhA* gene

^a^BAL, bronchoalveolar lavage

^b^The susceptibility to anti-TB drugs was determined by MGIT 960 system

^c^Mutations in *rpoB*, *katG* codon 315 and *inhA*-regulatory region were detected by GenoType MTBDR*plus* assay and/or PCR-sequencing of respective loci

^d^Represents a novel mutation not described previously

## Discussion

Kuwait is a low (24 cases per 100,000 population) TB incidence country [21]. Nearly 80% of all TB cases and > 90% of drug-resistant TB (including MDR-TB) cases in Kuwait occur in expatriate subjects mainly originating from TB endemic countries of South/Southeast Asia (such as Bangladesh, India, Pakistan and Philippines) [21, 27, 30]. Phenotypic DST of *M. tuberculosis* isolates in Kuwait during 2002–2010 was carried out simultaneously by BACTEC 460 TB system as well as by MGIT 960 system, however, BACTEC 460 TB system was discontinued on January 1, 2011 and phenotypic DST has been performed only by MGIT 960 system since 2011. Resistance rates for any first-line drug, INH, 2 or more drugs (excluding INH + RIF with/without additional resistance, polydrug resistance) and INH + RIF (with/without additional resistance, MDR-TB) were reported as 12.4, 9.1, 2.5 and 0.9%, respectively [21].

Rapid phenotypic methods (such as MGIT 960 system) are reliable for the detection of RIF-resistant strains with canonical *rpoB* mutations, however, they often fail to detect *M. tuberculosis* isolates with disputed *rpoB* mutations that exhibit low-level resistance to RIF [11–14]. In this study, we detected the presence of disputed *rpoB* mutations in 242 *M. tuberculosis* isolates phenotypically susceptible to RIF. While mutations in HSR-*rpoB* were detected by a line probe assay, mutation I572F in cluster II region of *rpoB* gene was detected by developing a simple agarose gel-based MAS-PCR assay. Previously, I572F mutation was detected either by PCR-sequencing or by a real-time PCR assay [15, 20, 29].

Our data showed that 4 of 242 (1.7%) RIF-susceptible isolates contained a disputed HSR-*rpoB* mutation while I572F mutation was not detected. The H526N mutation was found in 2 isolates while D516Y and S531C mutations were found in 1 isolate each. The D516Y and H526N mutations have previously been shown to increase RIF MIC of *M. tuberculosis* by 2–10 fold [13, 16, 17]. Two of 4 patients infected with *M. tuberculosis* strains carrying D516Y mutation in one study failed to respond to standard first-line treatment [17]. Similarly, 4 of 6 patients infected with *M. tuberculosis* strains carrying H526N mutation in another study either relapsed or failed treatment [19]. The S531C is a rare HSR-*rpoB* mutation and an isolate with S531C mutation was previously detected as RIF-resistant by BACTEC 460 TB system and/or agar dilution method [32]. However, the MGIT 960 system failed to detect RIF resistance in *M. tuberculosis* isolate (D4) with S531C mutation in this study. Interestingly, all 4 isolates with a disputed HSR-*rpoB* mutation in our study were polydrug-resistant (resistant to 2 or more drugs excluding RIF) strains resistant at least to INH (MDR-TB strains).

The occurrence of disputed *rpoB* mutations has been reported in few studies. The occurrence of disputed mutations in one study (carried out on *M. tuberculosis* isolates from Bangladesh and Democratic Republic of Congo) was found to be much higher (in 13.1% of all isolates from Bangladesh and in 10.6% of isolates from Democratic Republic of Congo), however, unlike our study, *M. tuberculosis* isolates from both these countries were cultured from retreatment (failure and relapse/reinfection after primary treatment) patients [12]. Two previous studies have reported the occurrence of disputed mutations among *M. tuberculosis* strains isolated from new TB patients [18, 20]. The data obtained in archived samples from Bangladesh showed that RIF resistance varied significantly among *M. tuberculosis* strains in different years (0.4% in 2005 versus 2.1% in 2010) and 7 of 1022 (0.7%) sputum samples contained *M. tuberculosis* with a disputed *rpoB* mutation [18]. Since the prevalence of RIF resistance varied significantly in different years and the fact that Bangladesh is among the top 30 high TB burden countries [1], the data may not be applicable for low TB incidence countries. The data from Australia showed that 5 of 214 drug-resistant isolates contained disputed HSR-*rpoB* mutations [20]. Among these 5 isolates, 4 isolates were monoresistant to INH and 1 isolate was monoresistant to PZA. None of 202 drug-susceptible *M. tuberculosis* isolates contained an HSR-*rpoB* mutation [20]. In contrast, all our isolates with a disputed HSR-*rpoB* mutation were polydrug-resistant strains resistant at least to INH but none of our 66 monodrug-resistant isolates contained an HSR-*rpoB* mutation.

Although the occurrence of RIF (and INH) resistance in 4 of 242 (1.7%) isolates appears to be higher than the reported occurrence [20] of MDR-TB in ~ 1% of *M. tuberculosis* isolates in Kuwait, the data should be interpreted with caution as the yearly occurrence of disputed *rpoB* mutations will likely be much lower (< 0.1%). This is because the proportion of polydrug-resistant strains was much higher (112 of 242, 46.3%) than normally reported (~ 3%) and the proportion of pansusceptible strains was much lower (64 of 242, 26.4%) than normally reported (~ 85%) among clinical *M. tuberculosis* isolates in Kuwait [21]. The rare occurrence of disputed *rpoB* mutations is probably also related to the fitness cost associated with these mutations. Isolates with mutations at codon 526 and 531 (except S531 L mutation) generally exhibit significantly decreased fitness which may lead to their removal from circulation and replacement by strains with greater fitness [33].

The clinical significance of disputed *rpoB* mutations is indicated by gene replacement studies and patients infected with such strains often fail treatment or relapse [17–20]. The clinical significance of disputed *rpoB* mutations in our study is also indicated by the following observations. i) All 4 isolates were resistant to INH and contained S315 T mutation in *katG* gene, an alteration that is strongly associated with acquisition of additional drug resistance leading to MDR-TB due to its minimal effects on fitness of tubercle bacilli [34, 35], ii) Molecular screening for EMB resistance showed that 3 of 4 (including 1 isolate phenotypically susceptible and 2 isolates phenotypically resistant to EMB) isolates contained M306 V mutation in *embB* gene that confers low-level resistance to EMB and is also strongly associated with MDR-TB phenotype [36–38], iii) Molecular screening for PZA resistance showed that 2 of 4 isolates (including 1 of 2 isolates for which phenotypic DST for PZA could not be performed) contained a *pncA* mutation that is either previously described in PZA-resistant strains [22, 23] or is highly suggestive of PZA resistance due to nonsynonymous mutation affecting the structure of pyrazinamidase, iv) Molecular screening for resistance conferring mutations showed that 3 of 4 isolates with a disputed *rpoB* mutation were actually resistant to three or all four first-line drugs (excluding streptomycin), and v) The isolates were genotypically unrelated as they were isolated at different (1 isolate each in 2005, 2010, 2011 and 2016) time points and all 4 isolates analysed by spoligotyping belonged to different *M. tuberculosis* lineages. Unfortunately, the final treatment outcome for the patients yielding *M. tuberculosis* isolates with disputed *rpoB* mutations was not available as the isolates were recovered from expatriate TB patients who were sent back to their respective country after initial treatment objective (sputum smear-negative status) was achieved. Similar observations regarding lack of final treatment outcome involving expatriate patients have also been recorded at other geographical locations [39].

Two other observations are noteworthy in our study. Among INH-resistant isolates, the frequency of *inhA-*RR mutations was higher than *katG315* mutations (42% versus 30%) in INH-monoresistant strains, however, this ratio was reversed in isolates with acquisition of additional phenotypic resistance to one more drug (15% versus 77% for isolates with INH + SM resistance and 8% versus 40% for isolates with INH + EMB resistance, Table 1). Furthermore, the *inh-*RR mutations were absent among INH-resistant isolates resistant to two other drugs. Our data support previous observations that fitness is not adversely affected by *katG315* mutation and they are more likely to acquire resistance to additional drugs [34, 35]. Our data also support recent observations showing that *katG315* mutations are significantly associated with additional resistance to SM and EMB and are more likely to cause unfavourable treatment outcome while *inhA*-RR mutations are mainly associated with additional resistance to SM only [40]. Secondly, our data also reiterate the limitations of phenotypic DST for EMB as isolates with low-level EMB resistance are often missed by these tests [25, 26] and phenotypic DST for PZA which often yields unreliable or no result [5, 6]. In this regard, one isolate (D2) for which phenotypic PZA susceptibility could not be determined contained a non-synonymous (R2P) mutation in *pncA*. To the best of our knowledge, this (R2P) mutation has not been described previously in the literature [22, 23]. Substitution of arginine by proline at amino acid position 2 is likely involved in conferring resistance to PZA as many non-synonymous *pncA* mutations described in the literature involve replacement of a wild-type amino acid with proline [22, 23]. Thus, molecular screening provides drug resistance profiles that help in proper management of MDR-TB patients as ineffective drugs are not included in drug regimens [3, 9, 41, 42].

Our study has a few limitations. (i) The MIC values of the isolates with a disputed *rpoB* mutation to RIF were not determined and (ii) The details of the patient’s treatment history and clinical outcome were not available as all 4 patients were expatriate subjects who were sent back to their respective country after initial treatment objective (sputum smear-negative status) was achieved.

## Conclusion

In conclusion, 4 of 242 (1.7%) RIF-susceptible *M. tuberculosis* isolates in Kuwait contained a disputed *rpoB* mutation and all 4 isolates were polydrug-resistant strains. The occurrence of an *rpoB* mutation in polydrug-resistant *M. tuberculosis* isolates resistant at least to INH (MDR-TB) suggests that polydrug-resistant strains from patients suspected to have MDR-TB should be checked for genotypic RIF resistance since the failure/relapse rates are nearly same in isolates with a canonical or disputed *rpoB* mutation.

## Abbreviations

DST: Drug susceptibility testing
EMB: Ethambutol
gMTBDR^+^: GenoType MTBDRplus assay
HSR-*rpoB*: 81-base pair hot-spot region of *rpoB* gene
INH: Isoniazid
*inhA*-RR: *inhA*-regulatory region
*katG315*: *katG* codon 315
MAS-PCR: Multiplex allele-specific-PCR assay
MDR-TB: Multidrug-resistant tuberculosis
MGIT: Mycobacteria growth indicator tube
MIC: Minimum inhibitory concentration
PZA: Pyrazinamide
RIF: Rifampicin
SM: Streptomycin
WHO: World Health Organization
XDR-TB: Extensively drug-resistant tuberculosis
Xpert: GeneXpert MTB/RIF assay
